# NEIGHBORHOOD SOCIAL INTEGRATION, SOCIAL NETWORK, AND COGNITION: DOES THE INTERACTION EFFECT MATTER?

**DOI:** 10.1093/geroni/igz038.118

**Published:** 2019-11-08

**Authors:** Jenhao Chen, Eileen Crimmins, Mengting Li, XinQi Dong

**Affiliations:** 1 Rutgers University, Camden NJ, United States; 2 University of Southern California, Los Angeles, California, United States; 3 Rutgers, The State University of New Jersey, New Brunswick, New Jersey, United States

## Abstract

Social integration of neighborhoods and social network properties are associated with better cognitive function but the two factors are often investigated separately. This study examines the interaction between neighborhood social integration and quantity and composition of social network on cognitive domains by analyzing Population Study of Chinese Elderly, a population-based epidemiological study of over 3000 US Chinese older adults aged 60 and above in Chicago metropolitan. Regression results show that larger network size, volume of contact and smaller proportion kin and proportion co-resident were associated with higher level of global cognition. Higher sense of community was associated with higher level of global cognition. The interaction term of volume of contact and neighborhood cohesion was negative and statistically significant, suggesting the protective effect of volume of contact may decrease in high cohesion neighborhoods. Similar moderation effects were observed in specific cognitive domains, including episodic memory, working memory and executive function.

